# Decline in age at menarche among Spanish women born from 1925 to 1962

**DOI:** 10.1186/1471-2458-9-449

**Published:** 2009-12-04

**Authors:** Anna Cabanes, Nieves Ascunce, Enrique Vidal, María Ederra, Ana Barcos, Nieves Erdozain, Virginia Lope, Marina Pollán

**Affiliations:** 1 Área de Epidemiología Ambiental y Cáncer, Centro Nacional de Epidemiología, Instituto de Salud Carlos III, Monforte de Lemos 5, 28029 Madrid, Spain; 2 Consortium for Biomedical Research in Epidemiology & Public Health (CIBER en Epidemiología y Salud Pública - CIBERESP). Instituto de Salud Carlos III, Madrid, Spain; 3 Instituto de Salud Pública, Sección de Detección Precoz, Bergamín 2, 31003 Pamplona, Spain

## Abstract

**Background:**

While the timing of reproductive events varies across populations, a downward trend in age at menarche has nevertheless been reported in most of the developed world over the past century. Given the impact of change in age at menarche on health conditions, this study sought to examine secular trends in age at menarche among women living in Navarre (Northern Spain) who participated in a population-based breast cancer screening programme.

**Methods:**

The study was based on 110545 women born from 1925 to 1962. Trends were tested using a linear regression model, in which year of birth was entered continuously as the predictor and age at menarche (years) as the response variable, using size of town and region of birth as covariates.

**Results:**

Among women born in Navarre between 1925 and 1962, age at menarche declined steadily from an average of 13.72 years in the 1925-1929 birth-cohorts to 12.83 years in the 1958-1962 birth-cohorts. Controlling for size of town or city of birth, age at menarche declined by an average of 0.132 years every 5 years over the period 1925-1962. This decline was greater in women born in rural versus urban settings. Trends were also different among regions of birth.

**Conclusion:**

We report a population-based study showing a downward trend in age of onset of menarche among Spanish women born in the period 1925-1962, something that is more pronounced among women born in rural settings and varies geographically.

## Background

Age of pubertal events is important individually, socially and culturally, and has a great impact on health conditions. In addition to the psycho-social and public health implications, early puberty is associated with increased risk of obesity, diabetes and cancer [1]. It is difficult to measure the stages of puberty accurately, and so to indicate the timing of puberty epidemiological studies often use age at menarche, as this event is well recalled many years later.

The time of reproductive events tends to vary across populations but a downward trend in age at menarche has been reported in most of the developed world over the last hundred years. The European Prospective Investigation into Cancer & Nutrition (EPIC) study found that mean age at menarche decreased among female participants born from 1912 to 1964 in nine European countries [2]. Other studies have also documented a trend towards earlier menarche in England [3], France [4], Israel [5], China [6] and the USA [7-9]. In Spain, cross-sectional studies have reported age at menarche at certain points in time [10-14] yet there are no data from population-based studies on trends in age at first menstrual period.

Although puberty follows a familial pattern and seems to have a strong genetic component [15], environmental factors also play a role. Nutritional status, chronic diseases, migration to a healthy environment, frequent infectious diseases, pollution and exposure to environmental oestrogens or endocrine disruptors can influence the endocrine milieu and affect pubertal development [1,16].

In view of the effects of advanced age at menarche on adolescent health, we considered it necessary to monitor menarche onset in the Spanish population. Accordingly, this study sought to examine secular trends in age at menarche among women living in the northern region of Navarre who attended a population-based breast cancer screening programme.

## Methods

### Study population

The study was based on 110545 women who participated a minimum of once in a population-based screening programme that was first launched in Navarre in September 1990 and targeted women aged 45 to 65 years (ages 45-69 years after 1998). The area covered by this programme is Navarre, one of Spain's 17 Autonomous Regions (*Comunidades Autónomas*). Navarre is a sparsely populated region and the majority of the local population is of Caucasian origin. According to the 2001 census, 33.1% of Navarre's inhabitants live in Pamplona, the regional capital, a figure that rises to 49.6% if the city's suburbs are included [17]. The entire population enjoys access to universal health care.

### Sampling procedure

All women coming within the designated age range and living in this autonomous region -regardless of nationality or legal status- are contacted by mail and given an appointment for the test. Subjects are screened every two years, unless the radiologist suggests an earlier mammography. All women residents of Navarre are screened under this programme, except those belonging to high-risk families, who are screened via specific programmes. A description of the principal indicators of the process and impact of the programme are available elsewhere [18].

### Data-collection

To study trends in age at menarche, we selected all Spanish-born screening programme participants in the period September 1990-December 2007, corresponding to women born between 1925 and 1962. Age at menarche, date and place of birth, and date of first interview were obtained from the Breast Cancer Screening Programme. In order to comply with Spanish legal and ethical requirements and guarantee confidentiality, data used for analysis purposes were rendered anonymous, with all identity tags being removed from the database and a random reference number being assigned to each subject.

Age at menarche in years was recorded at the first interview, based on the open question, "How old were you when your periods or menstrual cycles started?" The recalled age at menarche was confirmed at subsequent screenings.

### Analysis

Trends were tested using a linear regression model, in which year of birth was entered continuously as the predictor and age at menarche (years) as the response variable. By way of a marker of urban/rural economy, size of town of birth -available solely for women born in the Navarre Region- was used as a covariate to analyse differences in trends between urban and rural areas. Women in this subgroup were classified as having been born in a rural or urban environment, depending upon whether their town of birth had a population of over or under 10000 at their date of birth, according to the most contemporary census data [17]. The following were excluded: women with missing data on age at menarche or place of birth; and women who had reported an age at menarche of under 8 or over 20 years, inasmuch as these values were deemed extremely implausible.

Since menarche varies geographically, in a second analysis we analysed whether trend in age at menarche was different as between Autonomous Regions, using region of birth as a covariate.

## Results

The database contained data on 110545 women who had attended the breast cancer screening programme during the period September 1990-December 2007. Of these, some were excluded for one or more of the following reasons: they had been born outside Spain (n = 3121) or place of birth was missing (n = 169); data on age at menarche were missing (n = 330); reported age at menarche was under 8 years (n = 7) or above 20 years (n = 32). A total of 3518 women were excluded from the original database.

**Figure 1:**
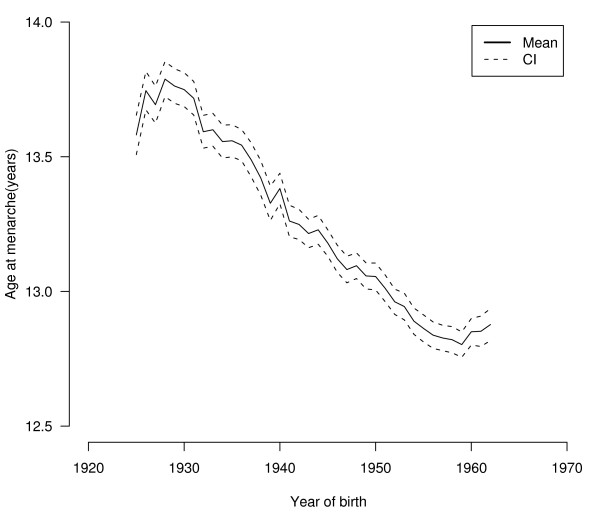
**Mean age at menarche and 95% confidence interval (CI) for women born in the period 1925-1962 (n = 107027)**.

Figure 1 depicts mean age at menarche by year of birth and its 95% confidence interval, and shows that, among women born between 1925 and 1962, age at menarche declined steadily, falling from an average of 13.71 years (n = 11220; 95% CI = 13.70, 13.75 years) in the 1925-1929 birth-cohorts to 12.83 years (n = 14745; 95% CI = 12.81, 12.85 years) in the 1958-1962 birth-cohorts.

A first regression model limited to women born in Navarre (n = 79396) was used to explore differences between urban and rural settings in trends in age at menarche. Rural/urban status was defined according to size of town of birth at subjects' date of birth. When this variable was included, the regression model improved significantly (F test; p < 0.001) (Table 1). When size of town of birth was controlled for, age at menarche decreased by -0.132 years every 5 years (95% CI = -0.137,-0.128) from 1925 to 1962. In addition to the confounding effect of size of town of birth, an interaction was observed between year of birth and this variable (likelihood ratio test; p < 0.001). Age at menarche declined by -0.136 years every 5 years on average (95% CI = -0.143,-0.131) among women born in towns of under 10000 inhabitants versus -0.120 every 5 years (95% CI = -0.130,-0.111) among women born in towns of over 10000 inhabitants (p < 0.001).

**Table 1:** Estimated age at menarche among women born in Navarre from 1925 to 1962, and percentage change over 5 years in cities or towns of over 10000 inhabitants compared to those of under 10000 inhabitants (n = 79396)

		Age at menarche first 5-year birth cohort (1925-1929)		Age at menarche last 5-year birth cohort (1958-1962)		5-year change		
**Size of city of birth**	**N**	**Mean**	**95%CI**	**Mean**	**95%CI**	**Years**	**95%CI**	**p**

**<10000 (reference)**	55085	13.73	13.70, 13.77	12.95	12.91, 12.99	-0.136	-0.143, -0.131	<0.001
>10000	24311	13.64	13.54, 13.74	12.82*	12.79, 12.86	-0.120*	-0.130, -0.111	<0.001

*Significantly different from reference group (p < 0.05)

A second analysis using all women born in Spain (n = 107027) showed that mean age at menarche among women born in the first part of our study period (1925-1929) differed among Autonomous Regions (Table 2). Whereas women born in La Rioja and Castile-León had significantly older ages at menarche than did those in Navarre, mean age at menarche was significantly lower in Castile-La Mancha and the Basque Country (p < 0.05). All other regions registered a mean age at menarche that was not significantly different to that in Navarre. In contrast, women born in Navarre during the last 5 years of the study period (1958-1962) had an older age at menarche than did women born in other regions of Spain (Table 2).

**Table 2:** Estimated age at menarche and percentage change over 5 years among women born from 1925 to 1962 in the different Autonomous Communities of Spain** (N = 107027)

Autonomous Community		Age at menarche First 5-year birth cohort (1925-1929)		Age at menarche Last 5-year birth cohort (1958-1962)		5-year change in age at menarche		
				
	N	Mean	95% CI	Mean	95% CI	Years	95% CI	p
**Navarre (reference)**	79396	13.72	13.69, 13.75	12.88	12.85, 12.90	-0.143	-0.143, -0.147	<0.001
Andalusia	4480	13.58	13.37, 13.79	12.52*	12.40, 12.64	-0.187	-0.187, -0.209	<0.001
Aragon	3109	13.57	13.38, 13.77	12.64*	12.48, 12.80	-0.138	-0.138, -0.163	0.757
Asturias	478	13.82	13.23, 14.41	12.41*	12.02, 12.79	-0.239	-0.239, -0.302	0.002
Basque Country	4004	13.36*	13.16, 13.55	12.74*	12.64, 12.83	-0.101	-0.101, -0.123	<0.001
Cantabria	428	13.59	12.92, 14.25	12.69	12.21, 13.17	-0.187	-0.187, -0.257	0.21
Castile-La Mancha	833	13.22*	12.77, 13.66	12.58	12.29, 12.88	-0.159	-0.159, -0.208	0.51
Castile-León	6036	13.91*	13.76, 14.07	12.98	12.87, 13.09	-0.170	-0.170, -0.189	0.005
Catalonia	683	13.46	13.01, 13.93	12.65	12.36, 12.93	-0.047	-0.047, -0.102	0.001
Extremadura	2448	13.71	13.40, 14.01	12.69*	12.53, 12.85	-0.161	-0.161, -0.191	0.235
La Rioja	2955	14.13*	13.92, 14.33	12.85	12.69, 13.02	-0.188	-0.188, -0.213	0.001
Galicia	825	14.13	13.68, 14.58	12.74	12.41, 13.07	-0.229	-0.229, -0.283	0.002
Madrid	786	13.93	13.40, 14.46	12.64	12.40, 12.88	-0.181	-0.181, -0.231	0.141
All women	107027	13.72	13.69, 13.75	12.83	12.81, 12.88	-0.145	-0.150, -0.145	<0.001

*Significantly different from reference group (p < 0.05)

**For the purpose of clarity, Autonomous Regions with N<400 are included in the analysis but not shown in this table

Age at menarche decreased by an average of -0.143 years every 5-years (95% CI = -0.143, -0.147) among women born in Navarre (F; p < 0.001) (Table 2). There was a statistical interaction between year of birth and Autonomous Region of birth (likelihood ratio test; p < 0.001). Compared to Navarre, the decline in age at menarche was significantly greater among women born in the regions of Andalusia (-0.187 years per 5-years), Asturias (-0.239 years), Castile-León (-0.170 years), La Rioja (-0.188 years) and Galicia (-0.229 years). Among women born in the Basque Country and Catalonia, however, the reduction was smaller (-0.101 and -0.047 every 5 years, respectively).

## Discussion

This is a population-based study that reports a secular trend towards an earlier age at menarche among Spanish birth cohorts from 1925 to 1962. The results are in agreement with previously published data from Spanish cross-sectional studies [11,13,14,19-21].

The study has several strengths, such as the large sample size and standardised data-collection methods throughout the study period. Moreover, being a population-based study, the results are truly representative of the population from which the study sample was drawn.

One limitation of our study is that age at menarche was self-reported at middle age, when women were admitted to the screening programme. Age at menarche recalled retrospectively could be a source of error, not only because of the fallible memory of the women surveyed but also because of inaccurate answers, i.e., restricted to age without specification of the precise month. Validation studies of recall of age at menarche at differing time-intervals suggest that long-term recall of age at menarche is quite valid [22-24]; individual women may not recall their age at menarche very accurately but studies generally report that some recall it earlier and others later than it really occurred, thereby rendering the mean value derived from recall reasonably accurate. However, other investigators report that age at menarche is often overestimated by 0.08 to 0.7 years when it is recalled in adulthood [24,25].

Other factors influencing the results and possibly inducing some degree of error are age at menarche and age of women at date of recall. Must et al. evaluated this error across menarcheal ages and found it to be non-linear [22-24]. Absolute error was smallest for both menarche that occurred earlier than the mean for the cohort (12.9 years) and menarche that occurred at older ages (>16 years).

To take in account any possible error resulting from age of women at recall, we performed a sensitivity analysis including only those women who had had their first interview before age 50 years. Even though the trends were less patent, age at menarche was nonetheless observed to decrease significantly in a manner similar to that seen in the main analysis, with the decline again being more pronounced among women born in small cities and towns. We did note, however, that declining trends were less marked among women born in large cities.

As a rule, later menarcheal age is characteristic of regions of low temperatures or high altitudes, whereas early menarcheal age tends to be common in areas with a benign climate, close to the coast [20]. However, as climate is associated with economic development, nutrition and lifestyle, it is difficult to evaluate the independent effect of climate on age at menarche. In our study, the decline in age at menarche varied, depending upon place of birth, though it did not follow any specific geographic pattern.

In the analysis restricted to women born in Navarre, menarche was found to occur earlier among women born in large cities but the decline in age at menarche was more marked in women born in small towns. Similar results are to be seen in other studies that report a strong decreasing trend in age at menarche among women in the highest percentile of age at menarche, in this case, women living in rural settings [26]. This indicates that differences between urban and rural areas in age at menarche may be disappearing, as borne out by the steeper decline in age at menarche observed among women born in rural towns.

Downward trends in menarcheal age have been reported for most of the developed world [2,27,28]. In European populations, mean age at menarche has been declining since the 1920s [2,3,26] but reports vary as to the magnitude of this decrease. Over similar periods, the highest falls were recorded in Israel and Belgium, namely, 0.237 and 0.187 years every 5 years respectively. In contrast, smaller declines of about 0.06-0.07 years every 5 years were recorded in the UK, USA, France and Greece. In Germany, Italy, The Netherlands and Sweden, age at menarche decreased at a similar rate to that in Spain, i.e., by around 0.15 years every 5 years.

In addition, most studies report that in nearly all populations secular trends in age at menarche levelled off around the time of the 1960 birth cohort [2,8,9,26,29-34].

The fall in menarcheal age among Spanish girls from 1925 to 1962 was probably caused by changes in nutritional, hygienic and health status. After the Civil War (1936-1939), living conditions gradually improved and the average weight of adolescent girls changed considerably [35].

Improved nutrition appears to play a key role, mainly through promotion of adipose-tissue growth rather than through the effect of specific macronutrients [36]. There is a large body of literature reporting that girls with higher body weight, higher body mass index, more body fat and greater height reach menarche earlier [31,37-42].

The mechanisms through which increased weight affects menarche onset are still unknown. One theory is the so-called "critical weight hypothesis", which suggests that girls need to reach a critical weight or height for menarche to occur, and that changes in lifestyle and dietary habits cause this critical weight to be attained at an earlier age [36,43]. Another hypothesis associates oestrogen's role in the initiation and progression of puberty with increased serum oestrogens in obese adolescent girls [44]. A third theory links a normal increase of insulin secretion and resistance to adiposity and the onset of puberty [45]. Finally, a fourth hypothesis, related to high body weight, highlights the permissive role of leptin in progression to puberty and maintenance of normal hypothalamic-pituitary-gonadal function [38,46]. In all likelihood, however, the key lies in the interaction among these various pathways.

A further possible cause of advanced onset of menarche is exposure to environmental oestrogens or endocrine disruptors. Menarche is linked to elevated oestrogen production, and environmental substances can exert this oestrogenic effect on animals and humans alike [47]. In this connection, we observed that in the more industrialised regions, such as Catalonia and the Basque Country, age at menarche was significantly lower than in other far less developed regions.

To sum up, we report a downward trend in the age of onset of menarche among Spanish women born between 1925 and 1962, something that is more pronounced among women born in small towns and varies geographically. As early menarche is an established risk factor for breast cancer, this may partially explain the increased incidence of breast cancer among Spanish premenopausal women in recent years [48].

Timing of puberty appeared to have stabilised in the last thirty years, until three studies reported a further advance in age at breast development among American and Danish girls [49-51]. Future research is called for to ascertain whether the same phenomenon is occurring among Spanish females and to achieve this, a key element will be active surveillance of the health indicators of adolescent girls at both an individual and population level.

## Conclusion

There has been a significant decline in age at menarche among Spanish women born in the period 1925-1962, a decline that is more pronounced among women born in small towns. Further studies are called for to ascertain the factors that explain these marked changes.

## Competing interests

The authors declare that they have no competing interests.

## Authors' contributions

AC: study design, analysis and interpretation of results, drafting the manuscript (corresponding author). NA: study design, data-collection and critical review of the manuscript (senior screening-programme researcher). EV: data-analysis and critical review of manuscript content. ME: study design, data-collection and critical review of the manuscript; AB: study design, data-collection and critical review of the manuscript. NE: study design, data-collection, compilation and checking of data, and critical review of the manuscript. VL: data-analysis; critical review of manuscript content. MP: design of research, analysis and interpretation of results, reviewing manuscript content (senior researcher of the team). All authors read and approved the final manuscript.

## Pre-publication history

The pre-publication history for this paper can be accessed here:

http://www.biomedcentral.com/1471-2458/9/449/prepub
